# Use of simultaneous traction over a halo ring to achieve reduction of a type 2 odontoid fracture for anterior odontoid screw fixation

**DOI:** 10.1016/j.ijscr.2019.09.028

**Published:** 2019-09-24

**Authors:** Sameh Abolfotouh, Don Moore

**Affiliations:** Department of Orthopaedic Surgery, University of Missouri, Columbia, MO, United States

**Keywords:** Bivector traction, Odontoid, Type 2, Anterior screws

## Abstract

**Introduction:**

Odontoid fractures are common. They represent 20% of all cervical trauma. There is a trend towards surgical stabilization. Fracture fixation with 1 or 2 anterior screws is standard operative treatment in younger population.

**Presentation of case:**

A case of type 2 odontoid fracture. The fracture was fixed initially with a halo vest temporarily. The patient was later treated operatively with anterior odontoid screw fixation. Reduction of the fracture was achieved using a bivector traction over a halo ring. The patient achieved an anatomical reduction and a rigid fixation.

**Discussion:**

Type 2 odontoid fracture is very controversial to treat. Several methods of fracture reduction have been described in the literature including Gardner-Wells Tongs and Mayfield head clamp. To our knowledge, fracture reduction with biverctor traction over a halo frame hasn’t been described before in the literature.

**Conclusion:**

Bivector traction over a halo ring can be used for intraoperative reduction of odontoid fracture.

## 1 Introduction

The treatment of odontoid fractures is very controversial. It represents one out of five of all the cervical trauma [1]. The commonest type of odontoid fracture is type 2 [1,2]. With the advances in imaging and surgical technologies, there is a trend toward primary operative stabilization [3]. In a recently published metanalysis, Posterior C1/2 fusion resulted in significantly higher fusion rates in the elderly [3]. However, the loss of motion at the C1/2 joint in younger patients should always be taken into consideration. Osteosynthesis with 1 or 2 screws by an anterior approach and fixation is standard operative treatment in younger patients with good bone quality. It was initially described by Bohler in 1982 [4]. It has a fusion rate of approximately 90% [[4], [5], [6]]. A crucial step in this technique is the reduction of the fracture and maintaining the reduced position. Reduction of such fracture can be challenging. Several reduction techniques can be used including Gardner- Wells Tongs and the Mayfield head clamp. We are presenting a case of type 2 odontoid fracture in which reduction of the fracture was achieved using simultaneous traction over a halo ring. This case has been reported in line with the SCARE criteria [7].

## 2 Case report

A 57 years old female patient presented to the Emergency department transferred from another hospital in a Miami J Collar after being involved in a motor vehicle condition with a complaint of neck pain. The patient was an unrestrained driver and was driving under the influence of Methamphetamine. On admission, her neurological examination was intact. She had full strength in all the muscle groups of both upper and lower extremities with intact sensory examination.

**Fig. 1:**
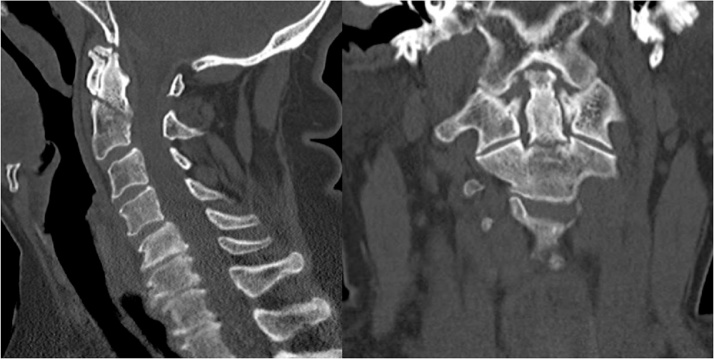
Saggiltal and coronal views of the cervical spine showing type 2 non-displaced odontoid fracture.

**Fig. 2:**
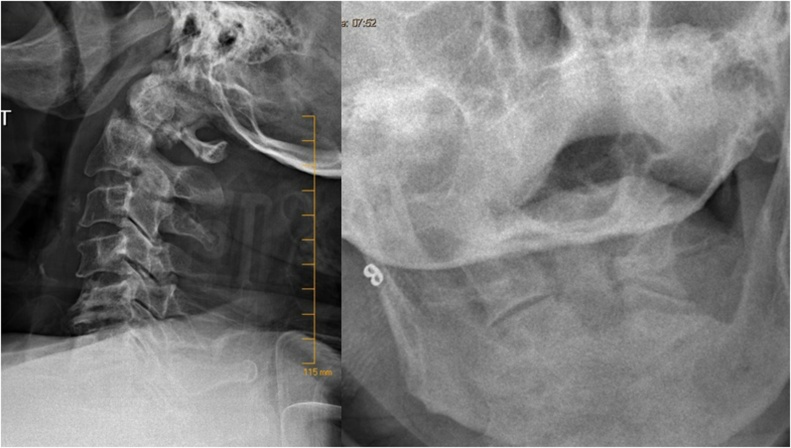
X-rays of the cervical spine (lateral and open mouth views) showing displaced and posteriorly angulated odontoid fracture.

A computed tomography was done at the initial hospital showed a non-displaced type 2 odontoid fracture (Fig. 1). Cervical radiographs that were done in our emergency department in an upright position showed that the fracture became displaced and angulated posteriorly (Fig. 2).

A decision of surgical intervention with anterior odontoid screw fixation was taken. Since the patient was under the methamphetamine intoxication and the fact that it is not a life saving surgery, the surgery was postponed for 5 days by the anesthesia team for fear of cardiac arrest during induction of general anesthesia.

**Fig. 3:**
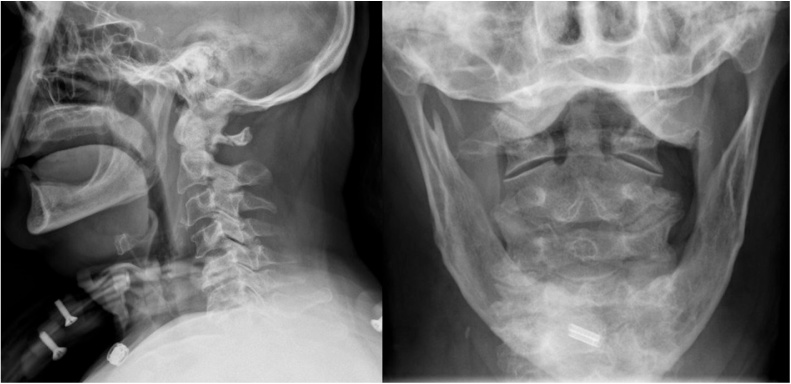
Post reduction X-rays of the cervical spine (lateral and open mouth views).

Considering the instability of the fracture, a temporary halo vest was applied in the emergency department to reduce the fracture and post reduction x rays were obtained (Fig. 3).

## 3 The technique

**Fig. 4:**
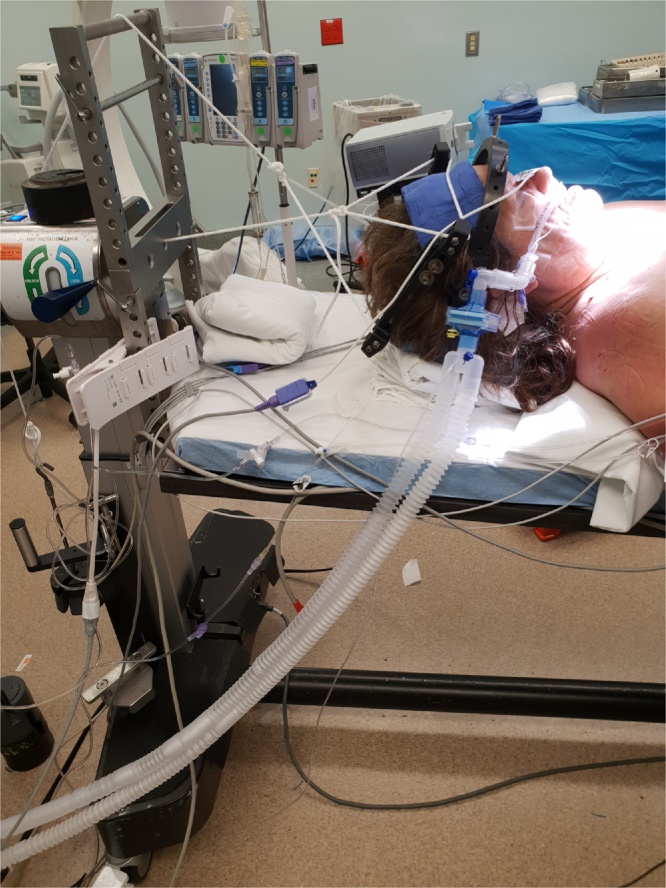
Illustration of the simultaneous traction technique with two traction ropes applied to the halo ring.

On the days of surgery, the halo vest was removed in the Operating room and the ring was kept in place. After introduction of general anesthesia using a glide scope in neutral alignment, the patient was transferred from the stretcher to the operating table with surgeon holding the halo ring to avoid any displacement of the fracture. Two traction ropes were applied to the halo ring. One rope applied to the posterior ring (Flexion rope) and the other one was applied to the mid portion of the anterior ring where the attachment to the halo vest rods were located (axial traction rope) (Fig. 4). The flexion rope travels from the posterior ring over the H-frame placed over the operating table creating a flexion vector. The axial traction rope extends from the middle of the anterior halo ring through the proximal end of the table and over the pulley creating a single axial traction vector. Flexion and axial traction at the fracture site was then achieved by applying sequential weights to both ropes to achieve adequate reduction of the fracture under fluoroscopy guidance. An anatomical reduction was achieved with 10 pounds on the flexion rope and 15 pounds on the axial traction rope.

**Fig. 5:**
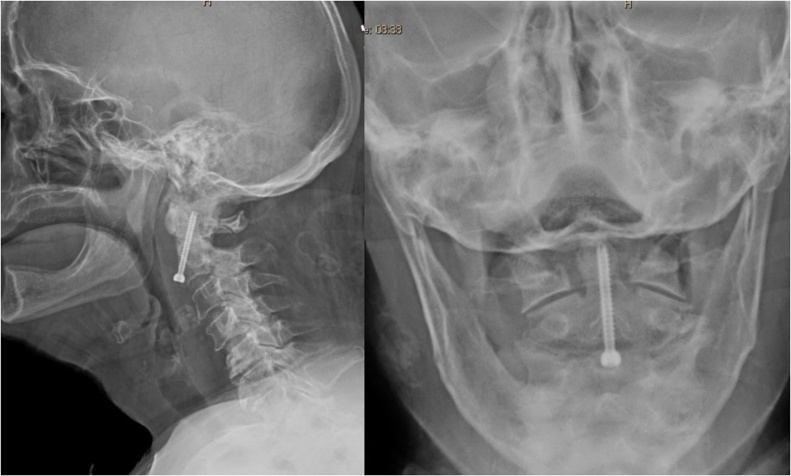
Final X-rays (lateral and open mouth views) showing maintained reduction of the fixation of the fracture.

The patient was then draped and prepared in sterile technique and a single 4 mm × 32 mm fully threaded anterior odontoid screw was placed through a Left-sided Smith-Robinson modified approach. Final x-rays showed maintenance of anatomical reduction and fixation of the fracture (Fig. 5).

## 4 Discussion

Odontoid fractures represent the most common cervical spine fracture accounting for 20% of them [8,9]. The majority of these fractures are type 2 which is very controversial to treat [10]. In 1988 Hadley described the high tendency to non-union of type 2A fractures [11].

Nakanishi [12] and Bohler [4] were the first to report odontoid fracture fixation with anterior odontoid screws. The fusion rate with this technique is reported to be approximately 90% [13].

Since their introduction in 1973 Gardner-Wells Tongs have become a popular method of cervical spine traction including the reduction of odontoid fractures [14,15]. A complication rate of 37.5%, consisting of loosening pins, asymmetric pins, and infection has been reported with their use in the literature [15]. On the other hand, the Mayfield head clamp is the most commonly used method of cervical traction and despite its popularity several reports of complications including pin loosening and scalp lacerations have been reported [[16], [17], [18]].

Bivector traction of the cervical spine has been described before by Riew et al. [19] for posterior cervical spine surgery. They used Gardner-Wells tongs for stabilizing the head and maintaining and improving the cervical sagittal alignment. To our knowledge, simultaneous traction over a halo ring for an odontoid fracture reduction and fixation has never been described before in the literature. The axial traction rope allows for distraction forces at the fracture site which allows for the disimpaction of the fractured fragment. The flexion rope allows for flexion forces at the fracture site which helps the reduction of the posteriorly displaced and angulated fracture fragment. In a case of anterior displacement of the cephalad fragment, we expect that a similar technique could be utilized with the flexion rope use in an extension manner instead.

## Sources of funding

No Source of funding.

## Ethical approval

Study is exempt from ethical approval in my institution.

## Consent

Written informed consent was obtained from the patient for publication of this case report and accompanying images. A copy of the written consent is available for review by the Editor-in-Chief of this journal on request”.

## Author’s contribution

Sameh Abolfotouh: Manuscript writing and Author of correspondence.

Don Moore: Manuscript writing.

## Registration of research studies

N/A.

## Guarantor

Don Moore, MD.

## Provenance and peer review

Not commissioned, externally peer-reviewed.

## Declaration of Competing Interest

The authors declare that they have no known competing financial interests or personal relationships that could have appeared to influence the work reported in this paper.
